# Impact of metabolic control on all-cause mortality in a nationwide cohort of patients with diabetes from Colombia

**DOI:** 10.3389/fendo.2023.1073833

**Published:** 2023-01-19

**Authors:** Carlos O. Mendivil, Mateo Amaya-Montoya, Juliana Alexandra Hernández-Vargas, Nathaly Ramírez-García, Lina Johana Herrera-Parra, Valentina Guatibonza-García, Camila Romero-Díaz, Agustín Pérez-Londoño, Lizbeth Acuña-Merchán

**Affiliations:** ^1^ School of Medicine, Universidad de los Andes, Bogotá, Colombia; ^2^ Endocrinology Section, Department of Internal Medicine, Fundación Santa Fe de Bogotá, Bogotá, Colombia; ^3^ Cuenta de Alto Costo, Fondo Colombiano de Enfermedades de Alto Costo, Bogotá, Colombia

**Keywords:** diabetes, metabolic control, complications, mortality, Latin America

## Abstract

**Objective:**

The magnitude of the mortality benefit conferred by good integral metabolic control in diabetes in not sufficiently known, especially among Latin American patients. We prospectively studied the association between sustained control of blood glucose (HbA1c<7%), systolic blood pressure (SBP) (<130 mmHg) and LDL (LDLc, <100mg/dL) and non-HDL (non-HDLc, <130 mg/dL) cholesterol, and death from any cause among all adult patients with diagnosed diabetes in Colombia.

**Methods:**

We retrospectively analyzed data from a nationwide, centralized, mandatory registry of all patients with diagnosed diabetes assisted by the Colombian health system between July 1, 2015, and June 30, 2019. We estimated the associations of sustained achievement of each goal, and of the joint triple goal (HbA1c + SBP + LDLc) with all-cause death. Associations were assessed after adjustment for sex, age, race, insurance type and BMI in multivariable logistic models.

**Results:**

We studied 1 352 846 people with diabetes. Sustained SBP (OR 0.42 [0.41-0.43]), HbA1c (OR 0.25 [0.24-0.26]) and LDLc (OR 0.28 [0.27-0.29]) control had strong negative associations with death. Moreover, among the 5.4% of participants who achieved joint, sustained metabolic control, the OR for death was 0.19 (0.18-0.21). Importantly, the impact of sustained, joint metabolic control was significantly smaller for patients of black race compared to other races (OR 0.31 [0.23-0.43] *versus* 0.18 [0.17-0.20], p-value for interaction <0.001), mostly at the expense of a smaller impact of LDLc control. The results were similar across body-mass index categories.

**Conclusions:**

Sustained and simultaneous metabolic control was associated with remarkably lower odds of death.

## 1 Introduction

The number of people with diabetes in Central and South America has reached the alarming figure of 32 million patients according to the 2021 version of the International Diabetes Federation (IDF) Atlas (1). This increase is expected to result not only in a heavy burden of disability and costs derived from chronic complications, but also in premature deaths. A large meta-analysis of data from the region found a 2.26‐fold increment in all-cause mortality among patients with diabetes compared to the general population (2). The relative growth in the risk of cardiovascular and renal mortality is even more dramatic.

However, the mortality risk imparted by diabetes can presumably be mitigated by metabolic control, measured as the achievement of basic treatment goals. These goals are frequently referred to as the ABC of diabetes (**A**1c, **B**lood pressure and **C**holesterol control). For example, in a long-term observational follow-up from the Diabetes Chronic Complications Trial - Epidemiology of Diabetes Interventions and Complications (DCCT/EDIC) study of patients with type 1 diabetes, mortality in the intensive treatment group (mean HbA1c 7% [53 mmol/mol]) was comparable to that of the general US population (3). Likewise, an observational follow-up study from the United Kingdom Prospective Diabetes Study (UKPDS) of patients with type 2 diabetes mellitus found that a 0.9% between-groups difference in HbA1c (4) during the active phase translated into 13% lower risk of death from any cause overall, and 27% lower risk among those allocated to intensive therapy with metformin (5).

Adequate blood pressure control is also a major determinant of mortality in diabetes. A meta-analysis including over 100 000 patients with type 2 diabetes concluded that each 10-mmHg lower systolic blood pressure was associated with a 13% reduction in total mortality, equivalent to an absolute reduction of three deaths/1000 patients/year (6). Control of low-density lipoprotein cholesterol (LDLc) also provides a mortality benefit. According to a Cholesterol Treatment Trialists´ Collaboration meta-analysis confined to over 18 000 patients with diabetes who took part in statin trials, all-cause mortality was reduced by 9% per each 38.6 mg/dL (1 mmol/L) decrement in LDLc (7). Further, the Steno-2 trial, despite its limited size, evidenced a very large mortality benefit (46% reduction) from the simultaneous control of the ABC goals in patients with type 2 diabetes over a 13-year follow-up (8).

Despite the clear mortality benefits of actively pursuing metabolic control in diabetes, its achievement remains markedly low. This holds true even in regions with advanced economies like the USA (9), Europe (10–13) and Japan (14). The situation is even more worrisome in Latin America, where the proportion of patients achieving the triple goal has been reported at 9.9% for the entire sub-continent (15) and 25.4% for Colombia (16).

Over the recent years, diabetes seems to be on the rise in Colombia. Despite a scarcity of large population-based data, the prevalence among adult urban residents was estimated at about 10% in 2018 (1, 17). In addition, diabetes holds an important place as a cause of mortality in Colombia (8^th^ place (18)) and countries of a similar sociodemographic and cultural background.

Within this context, we aimed to assess the association between metabolic control and total mortality in a nationwide registry of all patients with diagnosed diabetes served by the Colombian Health System, between 2015 and 2019. We also examined how this association varied according to race and body-mass index (BMI) categories. The exploration and documentation of the impact of metabolic control is a key input to support the design of public health interventions aimed at improving life expectancy among patients with diabetes.

## 2 Subjects and methods

### 2.1 Data sources

The Colombian National Registry of Chronic Kidney Disease (NRCKD) is a database of all people with diagnosed diabetes, hypertension or chronic kidney disease who have been assisted by the Colombian Health System. The NRCKD is managed by the High-Cost Diseases Colombian Fund (“*Fondo Colombiano de Enfermedades de Alto Costo*” - CAC in Spanish) and has been operating since 2008 through a resolution from the Colombian Ministry of Health (19). Each new registry cycle starts on July 1 of a year, and ends on June 30 of next year. The NRCKD is a passive registry with a national scope because almost the totality of the population is affiliated to the national healthcare system (19), and insurers are mandated by law to report all eligible patients to the registry (19).

As previously described (16), for each new case entering the NRCKD an initial registration is completed; after which data are updated every year. Each data point registered in the database corresponds to the last measurement of that variable within the observation period. The NRCKD undergoes a data validity auditing process with several steps. The first step involves the use of an algorithm to identify mistakes in the reporting process. Then, an experienced team compares the reported information with clinical records by a well-established data monitoring process in a representative sample of cases stratified by hypertension, diabetes, and CKD status (20). If any inconsistency is identified, real data from clinical records are captured.

### 2.2 Eligibility and variables

We retrospectively analyzed data on all adults with diabetes reported to the NRCKD between July 1^st^, 2015 and June 30^th^, 2019. For each year of the study, we excluded people aged <18 at the start of the study year. The presence of a diagnosis of diabetes or hypertension was analyzed as reported to the NRCKD (Y/N as defined by the treating physician).

Metabolic control goals were defined according to recommendations by the International Diabetes Federation, the American Diabetes Association, and the Latin American Diabetes Association - ALAD (21–23). Treatment goals were HbA1c <7% (<53 mmol/mol), systolic blood pressure (SBP) <130 mmHg, and LDL cholesterol (LDLc) <100 mg/dL. We also analyzed non-HDL cholesterol (non-HDLc) below 130 mg/dL as an exposure. The joint triple goal was HbA1c <7% (<53 mmol/mol) plus SBP <130 mmHg plus LDLc <100 mg/dL.

Data from the NRCKD were used to classify participants in terms of age, sex, race or ethnic group, and insurance status. The database also contains data on weight and height, BMI was classified as recommended by the World Health Organization (24). Plasma creatinine values were used to calculate the estimated glomerular filtration rate (eGFR) using the Modified Diet for Renal Disease (MDRD) equation, which has been found to be more accurate than other equations among patients with diabetes (25). Based on eGFR, CKD stages were defined as follows: stage 1: GFR ≥ 90 mL/min; stage 2: GFR: 60-<90 mL/min; stage 3: GFR: 30-<60 mL/min; stage 4: GFR 15-<30 mL/min and stage 5: GFR: <15 mL/min.

The Colombian health system has three health insurance types: Third-party payer (“*régimen contributivo*”), run by private insurers (*“Empresas Promotoras de Salud” – EPS*); state-run insurance (“*régimen subsidiado*”), run by a different type of insurer (mostly “*Administradoras de Régimen Subsidiado - ARS*) and a special/exceptional health system for the security forces and employees of some public universities (*régimen especial*/*régimen de excepción*) (26). We studied insurance type using these three categories.

For the effects of this study, we collapsed the NRCKD race categories “Raizal”, “Palenquero” and “Black, Mulatto, Afro-Colombian or Afro-descendant” into a single category called “black” and analyzed self-reported race as black vs. all others. We made this decision because very few individuals (<1% in any given year) identified themselves as belonging to one of the other race categories (indigenous or Roma).

### 2.3 Data analysis

For descriptive analyses of baseline clinical and demographic characteristics, quantitative variables are presented as means and standard deviations, categorical variables as absolute and relative frequencies. For all analyses, the main outcome was death from any cause, inferred from the variable “date of death”, reported by insurers to the NRCKD as the date registered in the death certificate if the patient died during the observation year, or as missing if the patient was still alive. This information was double-checked against the data warehouse of the social protection integrated information system (SISPRO) of the Colombian Ministry of Health and Social Protection. In the case of a discrepancy, the Colombian database of death certificates was consulted.

The association between metabolic control goals as explanatory variables and death from any cause as outcome was evaluated using multivariable logistic regression models. There was a set of potentially confounding variables adjusted for in all models, including sex, age, race, insurance type and BMI. Additionally, we adjusted for the variables representing goals other than the one being evaluated. Thus, in models to evaluate the association between HbA1c goal and mortality, we adjusted for the basic set of confounders, plus hypertension status and non-HDLc. When SBP was the main exposure, we adjusted for the basic confounders plus HbA1c and non-HDLc. When one of the plasma lipids was the exposure, we adjusted for basic confounders plus hypertension status and HbA1c. In models evaluating the triple metabolic goal, we only adjusted for the basic confounders.

We performed two types of analyses, a first group considering as independent variable baseline metabolic control, and a second group modelling the effect of sustained metabolic control throughout the complete study period. The set of confounders being adjusted for was identical in both groups of analyses. Additionally, we performed stratified analyses to explore how metabolic control was related to mortality in subgroups defined by race (black vs other), and BMI category (normal, overweight or obesity). Interactions were tested by the significance of the regression coefficient associated to a multiplicative interaction term between goal achievement status and the stratification variable. All associations are expressed as Odds Ratios (OR) with 95% confidence intervals. To explore the robustness of findings and sort out reverse causation, we performed a sensitivity analysis of metabolic control and mortality excluding participants with CKD stages 4 or 5 at baseline. All analyses were performed at a 5% significance level, and all reported significance tests are two-tailed. Statistical analyses were performed in Stata version 17 (StataCorp LP, College Station, Texas, USA).

### 2.4 Ethical considerations

This research was based on anonymized secondary data sources and did not include any private information that could make any subject identifiable. To protect privacy, data were anonymized through the use of a database-specific individual ID. Because the study involved only secondary retrospective analyses of an anonymized database, it did not qualify as human subjects research as was exempted from IRB review. Confidentiality was guaranteed throughout the information processing (reporting, managing, and analysis).

## 3 Results

We studied 1 352 846 adults with diagnosed diabetes from the NRCKD, mean age was 60.8 years, 42.3% were male, 6.6% reported their race as black and almost four of every five patients were overweight or obese. Average systolic and diastolic blood pressure levels were within the normal range, while HbA1c was well above 7%. Mean LDLc and non-HDLc also exceeded recommended thresholds. Most participants were in CKD stage 1, and had a urinary albumin excretion rate (UAER) below 30 mg/g of urinary creatinine or 20 mg/dL. Diagnosed hypertension was highly prevalent (**Table 1**). Follow-up duration was four years in 59.0% of participants, three years in 15.4%, two years in 12.7%, and one year in 12.9%. As compared to men, a larger proportion of women had a BMI in the obesity range.

**Table 1:** Baseline characteristics of study participants.

Variables	Men (n=572 383)	Women (n=780 463)	Total (n=1 352 846)
Age (yrs)	60.2 (13.6)	61.3 (14.0)	60.8 (13.9)
Age group (%)			
<40	7.2	6.8	7.0
40-49	13.9	12.0	12.8
50-59	25.8	25.4	25.6
60-69	27.5	27.2	27.3
70-79	17.8	18.9	18.5
≥80	7.7	9.7	8.9
Health insurance (%)			
Third-party payer	70.6	59.8	64.4
State	27.4	38.9	34.0
Special/Exceptional	2.0	1.3	1.6
Race (%)			
Black	6.3	6.8	6.6
Other	93.7	93.2	93.4
n for BMI	567 768	772 671	1 340 439
BMI (Kg/m^2^)	27.6 (4.8)	28.3 (5.6)	28.0 (5.3)
BMI category (%)			
Normal weight	21.2	20.9	21.1
Overweight	31.3	27.2	28.9
Obesity	47.5	51.9	50.0
n for blood pressure	553 722	757 425	1 311 147
SBP (mmHg)	124.1 (14.3)	124.4 (14.6)	124.3 (14.5)
DBP (mmHg)	76.7 (8.9)	76.6 (8.9)	76.7 (8.9)
n for HbA1c	444 573	587 212	1 031 785
HbA1c (% of total Hb)	7.55 (2.12)	7.45 (2.05)	7.49 (2.08)
HbA1c (mmol/mol)	59	58	58
n for blood lipids	449 351	620 820	1 070 171
Non-HDLc (mg/dL)	139.1 (46.1)	146.1 (47.0)	143.2 (46.8)
LDLc (mg/dL)	105.5 (38.3)	112.6 (40.0)	109.6 (39.5)
CKD stage (%)			
1	64.1	59.3	61.3
2	26.0	28.4	27.4
3A	5.6	8.0	7.0
3B	2.2	2.7	2.5
4	0.8	0.8	0.8
5	1.3	0.8	1.0
Urinary albumin excretion (%)			
<30 mg/g or <20 mg/dL	69.1	76.2	73.2
30-300 mg/g or 20-199 mg/dL	25.0	19.8	22.1
>300 mg/g or ≥200 mg/dL	5.9	4.0	4.8
Hypertension (%)	62.7	69.4	66.6

The year 2016 corresponds to data registered between July, 2015 and June, 2016, and so on. Data are means (SD) unless indicated otherwise. BMI, Body mass index; SBP, Systolic blood pressure; DBP, Diastolic blood pressure; LDLc, LDL cholesterol; non-HDLc, non-HDL cholesterol; CKD stage was classified according to the KDIGO classification.

### 3.1 Baseline metabolic control and mortality

There were 107 839 deaths over the entire follow-up (total cumulative mortality 7.97%). The variable whose baseline control was most strongly associated with lower mortality was a SBP<130 mmHg, an association that persisted and actually became stronger after multivariable adjustment (OR 0.72, 95% CI 0.71-0.74)). Glycemic control with HbA1c<7% at baseline was associated with only 3% lower odds of mortality in univariate analysis, but after adjustment for confounders the negative association grew stronger (18%, 95% CI 17-19%). Patients who achieved the joint triple goal at baseline had significantly lower mortality (OR 0.85, 95% CI 0.83-0.87).

### 3.2 Sustained metabolic control and mortality

**Figure 1:**
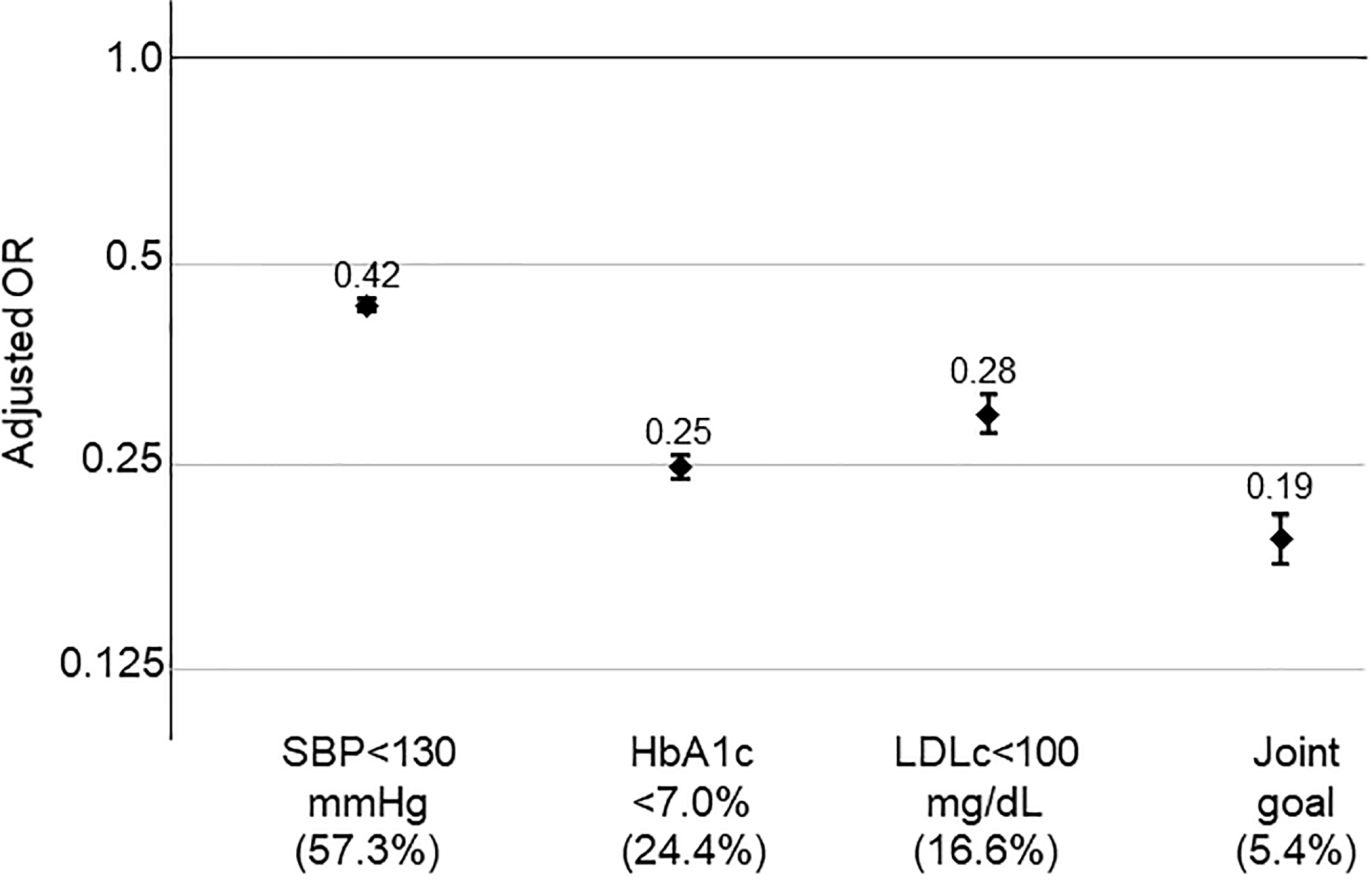
Association between sustained metabolic control and mortality. Data are odds ratios compared to participants not reaching sustained achievement of each goal, adjusted for sex, age, race (black vs other), insurance type and baseline BMI. Error bars represent 95% confidence intervals. Numbers in parentheses represent the percentage of study participants who sustained control of each goal over study follow-up.

The effect of sustaining basic diabetes treatment goals largely exceeded that of achieving them only at baseline. In models adjusted by sex, age, race, insurance type and baseline BMI, sustained SBP and HbA1c control were associated with 58% lower and 75% lower odds of death, respectively (**Figure 1**). Sustained control of LDLc under 100 mg/dL also showed a strong negative association with mortality (OR 0.28 [0.27-0.29]). When we used the stricter 70 mg/dL cLDL goal, sustained LDLc control was associated with 75% lower odds of all-cause mortality (OR 0.25, 95% CI 0.23-0.27). When the three metabolic goals were achieved and sustained, the decrease in the odds of mortality was 81% (79-82%) (**Figure 1**). Unfortunately, only 5.4% of study participants both achieved and sustained this degree of metabolic control. When a non-HDLc <130 mmHg substituted LDLc<70 mg/dL in the triple goal, the results were quite consistent, maintenance of this joint goal was associated with an adjusted OR for mortality of 0.19 (95% CI 0.17-0.21). Sustained control of albuminuria (urinary albumin excretion rate<30mg/g creatinine or <20 mg/L), was associated with a multivariable-adjusted OR for total mortality of 0.26 (95% CI: 0.24-0.27).

### 3.3 Sustained metabolic control and mortality, by race

The positive effect of SBP control did not differ by between patients of black race and those of other races (*p-value* for the interaction 0.32). By contrast, the beneficial effect of sustained glycemic control was significantly smaller for patients of black race (OR 0.39, 95% CI 0.34-0.45 for black race; OR 0.24, 95% CI 0.25-0.25 for other races, *p-value* for interaction <0.001) (**Table 2**). However, the largest difference according to race existed in the relationship of sustained lipid control and mortality, the impact being much larger for patients of non-black race. The reduction in the OR for sustained control of non-HDLc was 30% for black patients and 73% for patients of other races (*p-value* for interaction<0.001). In the case of sustained LDLc control, the figures were 59% lower odds for black patients, and 73% lower odds for patients of other races (*p-value* for interaction <0.001). In consequence, the reduction in the odds of death with sustained achievement of the triple goal was 69% for black patients, and 82% for patients of other races (**Table 2**).

**Table 2:** Association between sustained metabolic control and mortality, by race.

	Race						
	Black			Other			
Sustained metabolic control goal	aOR (95% CI)	n for model	Events for model	aOR (95% CI)	n for model	Events for model	*p-value* for interaction
SBP < 130 mmHg †	0.42 (0.38-0.46)	58 400	2 373	0.42 (0.41-0.43)	771 052	35 273	0.32
HbA1c < 7.0% ‡	0.39 (0.34-0.45)	52 802	2 398	0.24 (0.23-0.25)	715 101	36 082	<0.001
Non-HDLc < 130 mg/dL §	0.70 (0.57-0.85)	48 499	2 174	0.27 (0.26-0.29)	651 802	34 389	<0.001
LDLc < 100 mg/dL §	0.41 (0.35-0.48)	54 822	2 382	0.27 (0.26-0.28)	704 675	34 960	<0.001
Joint SBP, HbA1c and LDLc goal ¶	0.31 (0.23-0.43)	56 036	2 801	0.18 (0.17-0.20)	753 074	43 779	0.003
Joint SBP, HbA1c, LDLc and BMI goal *	0.35 (0.21-0.59)	58 763	3 060	0.22 (0.19-0.25)	799 878	48 556	0.10

aOR, Adjusted Odds Ratio. † Adjusted for core confounders (sex, age, race [black vs other], insurance type and BMI), baseline HbA1c and baseline non-HDL cholesterol. ‡ Adjusted for core confounders, baseline non-HDL cholesterol, and hypertension. § Adjusted for core confounders, baseline HbA1c, and hypertension status. ¶ Adjusted only for core confounders, except BMI. * Adjusted only for core confounders, except BMI.

### 3.4 Sustained metabolic control and mortality, by BMI category

There was no significant difference in the association between sustained SBP control and mortality for patients with a BMI in the normal, overweight or obesity range (*p-value* for interaction 0.54, **Table 3**). Conversely, sustained glycemic control was most negatively associated with mortality among patients with obesity (*p-value* for interaction <0.001). While sustained control of LDLc had a similar impact in all BMI categories, non-HDLc control also had a greater impact in the obesity category (*p-value* for interaction 0.001). Remarkably, the effect of sustained joint metabolic control on mortality was similar across BMI categories, with no significant interaction (**Table 3**).

**Table 3:** Association between sustained metabolic control and mortality, by body-mass index category.

	BMI category									
	Normal weight			Overweight			Obesity			
Sustained metabolic control goal	aOR	n for model	Events for model	aOR	n for model	Events for model	aOR	n for model	Events for model	*p-value* for interaction
SBP < 130 mmHg†	0.39 (0.37-0.41)	172 555	12 024	0.46 (0.45-0.48)	269 179	13 674	0.40 (0.39-0.42)	387 708	11 948	0.54
HbA1c < 7.0% ‡	0.26 (0.24-0.28)	158 861	12 708	0.28 (0.26-0.29)	249 311	14 100	0.21 (0.20-0.22)	359 731	11 672	<0.001
Non-HDLc < 130 mg/dL §	0.32 (0.28-0.37)	139 739	11 804	0.38 (0.33-0.44)	222 025	13 365	0.27 (0.25-0.30)	338 516	11 394	0.001
LDLc < 100 mg/dL §	0.28 (0.26-0.30)	160 056	12 491	0.29 (0.27-0.31)	240 728	13 302	0.28 (0.26-0.30)	358 699	11 549	0.98
Joint SBP, HbA1c and LDLc goal ¶	0.17 (0.14-0.20)	170 598	15 838	0.23 (0.20-0.27)	260 990	16 724	0.18 (0.16-0.21)	377 500	14 018	0.54

aOR, Adjusted Odds Ratio (95% CI). † Adjusted for sex, age, race (black vs other), insurance type, baseline HbA1c and baseline non-HDL cholesterol. ‡ Adjusted for sex, age, race (black vs other), insurance type, baseline non-HDL cholesterol, and hypertension status. § Adjusted for sex, age, race (black vs other), insurance type, baseline HbA1c and hypertension status. ¶ Adjusted for sex, age, race (black vs other) and insurance type.

### 3.5 Sensitivity analyses excluding advanced CKD at baseline

Given that individuals with advanced CKD may experience reductions in blood pressure, glycemic levels and/or plasma lipids towards the end of life (leading to the so-called reverse causation problem), we performed a sensitivity analysis of sustained goal achievement and mortality excluding participants at CKD stages 4 or 5 at baseline. The results were generally consistent with those for the complete study sample. The OR for mortality according to sustained control of each variable were 0.50 (0.49-0.51) for SBP, 0.25 (0.24-0.26) for HbA1c, 0.27 (0.26-0.29) for LDLc, and 0.21 (0.19-0.23) for the triple goal (**Table 4**).

**Table 4:** Association between metabolic control and mortality, excluding patients in CKD stages 4 or 5 at baseline.

Sustained treatment goal	Adjusted OR	n for adjusted model	Events for adjusted model
SBP < 130 mmHg†	0.50 (0.49-0.51)	812 077	32 387
HbA1c < 7.0%‡	0.25 (0.24-0.26)	752 097	33 744
Non-HDLc < 130 mg/dL§	0.32 (0.30-0.34)	687 398	32 541
LDLc < 100 mg/dL§	0.27 (0.26-0.29)	744 879	33 399
Joint SBP, HbA1c and LDLc goal¶	0.21 (0.19-0.23)	790 844	40 356
Joint SBP, HbA1c, LDLc and BMI goal*	0.26 (0.23-0.31)	839 558	45 008

† Adjusted for core confounders (sex, age, race [black vs other], insurance type and BMI), baseline HbA1c and baseline non-HDL cholesterol. ‡ Adjusted for core confounders, baseline non-HDL cholesterol, and hypertension. § Adjusted for core confounders, baseline HbA1c, and hypertension status. ¶ Adjusted only for core confounders. * Adjusted only for core confounders, except BMI.

## 4 Discussion

In this large scale, nationwide retrospective study of people with diabetes in Colombia, we found a very strong negative association between good metabolic control and death from any cause. Sustained control of SBP was associated with up to 58% lower odds of death, HbA1c control with 75% lower odds, and LDLc control with 72% lower odds. A powerful finding was that the continuous achievement of the triple metabolic goal was accompanied by an 81% reduction in total mortality. The inclusion of either LDLc or non-HDLc as lipid parameter in the joint goal produced very similar results.

Several trials have compared more versus less strict blood pressure control in diabetes, most notably the UKPDS-38 (27) and ACCORD-BP (28) trials. Even though UKPDS-38 found significant reductions in total diabetes complications, the effect on total mortality was not significant. Likewise, in ACCORD-BP there was a significant reduction in strokes with tighter SBP control, but not a mortality effect, although the study was underpowered for total mortality. Nonetheless, the previously mentioned meta-analysis of blood pressure control trials in diabetes found a continuous negative relationship between achieved SBP and mortality (6). Our results support the idea that in usual practice conditions, achievement of this SBP goal does translate into lower risk of death among patients with diabetes.

Concerning glycemic control, the UKPDS documented a monotonic negative relationship between achieved HbA1c and adjusted total mortality rate (29). Later studies have found this association to be strongest among patients with low glucose variability (30). We had the very interesting finding that sustained glycemic control had a negative relationship with mortality four times larger than just baseline control. In a recent observational study in Israel, patients newly diagnosed with type 2 diabetes who experienced an early reduction followed by a sharp, progressive increment in HbA1c had an 83% higher risk of death over a five-year period, relative to those with stable, controlled HbA1c levels (31). These results highlight the importance of active HbA1c monitoring and avoidance of clinical inertia in diabetes management. Our results were markedly different for baseline *versus* continued lipid control, the protective effect being present only for the sustained control of LDLc or non-HDLc. A similar problem had been reported in a nationwide study of the association between statin use and cancer mortality in Denmark (32). Our results emphasize the importance of strict and continuous LDL and non-HDL control among people with diabetes.

Next to the impressive effect of joint and sustained metabolic control, an alarming result was that only 5.4% of patients achieved this fundamental treatment objective. Globally, better metabolic control has been held responsible for recent downward trends in mortality among patients with diabetes (33), despite the increase in diabetes prevalence (34). Recent analyses of data from the U.S. Veteran Administration warehouse found that control of each additional ABC goal was associated with a significant improvement in mortality (35). Thorough metabolic control is feasible and provides substantial benefits, every possible effort should be made to achieve it in all patients with diabetes.

Data from several countries indicate that patients of black race have increased rates of diabetes complications (36). In the United States, African American patients with diabetes are four times more likely to have end-stage renal disease than non-Hispanic whites (37), a difference that can be explained only partially by disparities in socioeconomic status or access to healthcare (36). In our study, black race was an important modifier of the relationship between metabolic control and mortality. Compared to other races, patients of black race derived the same benefit from sustained SBP control, but significantly less benefit from achievement of the HbA1c, non-HDLc or LDLc goals, or of the joint triple goal. The Heart Outcomes Prevention Evaluation (HOPE)-3 trial, found no evidence of a differential effect of LDLc control with rosuvastatin on major cardiovascular events in patients of black race compared to other races (38). In fact, in the very large ALLHAT-LLT study, patients of black race seemed to derive *larger* coronary heart disease reductions from LDLc control (39). Thus, it might be that in our particular population a lower LDL among people of black race acts as a marker of other risk factors for mortality like social or economic deprivation. This hypothesis, however, will need to be tested in future studies.

We also found that good metabolic control provides large benefits in patients of *any* BMI. Importantly, our central findings remained essentially unaltered in sensitivity analyses excluding patients in baseline CKD stages 4 or 5.

### 4.1 Study strengths and limitations

Our study is based on a large, nationwide, centrally administered database of compulsorily reported and constantly audited data. In addition, the primary outcome of death from any cause is verified against official government sources. Limitations of the study include its relatively short follow-up for metabolic control to impact mortality. Also, we could not differentiate between patients with type 1 or type 2 diabetes, and metabolic control may have a numerically different impact in these two patient populations. In addition, the NRCKD database does not collect data on current medications, except for angiotensin-converting enzyme inhibitors and angiotensin receptor blockers. Arguably, however, most of the effects of antidiabetic, antihypertensive or lipid lowering drugs should be manifest in the values of SBP, HbA1c or blood lipids, which we did analyze. As in most registry-based studies of chronic diseases, the exact date of start of diabetes was not known, so we could not include diabetes duration as a covariate in our models.

In conclusion, our results showed that successful control of fundamental variables has a strong negative association with mortality in diabetes. Despite the existence and availability of the lifestyle and pharmacological means to attain these goals, they are still not being achieved by the overwhelming majority of patients. Widespread pursuit of tight metabolic control may yield large benefits in terms of mortality in diabetes.

## Data availability statement

The raw data supporting the conclusions of this article will be made available by the authors, without undue reservation.

## Ethics statement

Ethical review and approval was not required for the study on human participants in accordance with the local legislation and institutional requirements. Written informed consent for participation was not required for this study in accordance with the national legislation and the institutional requirements.

## Author contributions

CM: Conceptualization, Data curation, Formal analysis, Funding acquisition, Methodology, Supervision, Visualization, Writing - original draft, Writing - review & editing. MA-M: Data curation, Formal analysis, Methodology, Visualization, Writing - original draft; Writing - review & editing. JH-V: Conceptualization, Data curation, Investigation, Methodology, Project administration, Resources, Software, Writing - original draft; Writing - review & editing. NR-G: Conceptualization, Data curation, Investigation, Methodology, Project administration, Resources, Software, Writing - original draft; Writing - review & editing. LH-P: Conceptualization, Data curation, Investigation, Methodology, Project administration, Resources, Software, Writing - original draft; Writing - review & editing. VG-C: Data curation, Formal analysis, Methodology, Writing - original draft; Writing - review & editing. CR-D: Data curation, Formal analysis, Methodology, Writing - original draft; Writing - review & editing. AP-L: Data curation, Formal analysis, Methodology, Writing - original draft; Writing - review & editing. LA-M: Conceptualization, Data curation, Investigation, Methodology, Project administration, Resources, Software, Writing - review & editing. All authors contributed to the article and approved the submitted version.
